# Construct validity, test–retest reliability, and responsiveness of the Arabic version of the upper limb functional index

**DOI:** 10.1186/s12891-023-06969-8

**Published:** 2023-10-31

**Authors:** Yousef A. Albahrani, Ali M. Alshami

**Affiliations:** 1 Department of Rehabilitation, Prince Mohammed Bin Abdulaziz Hospital, Riyadh, Saudi Arabia; 2 Department of Physical Therapy, Imam Abdulrahman Bin Faisal University, Dammam, Saudi Arabia

**Keywords:** Psychometric properties, Reliability, Validity, Responsiveness, Outcome measure

## Abstract

**Background:**

The upper limb functional index (ULFI) is a widely used self-report outcome measure questionnaire with robust psychometric properties to assess the upper limb musculoskeletal disorders (UL-MSDs). This study aimed to investigate the psychometric properties of the Arabic version of ULFI (ULFI-Ar).

**Methods:**

In this observational study, 139 patients (87 male, 52 females with mean age of 38.67 ± 13.04 year) with various UL-MSD’s, completed the ULFI-Ar, Disability of Arm, Shoulder, and Hand questionnaire (DASH-Arabic), and numeric pain rating scale (NPRS-Arabic). All participants determined the factor structure, and the construct validity. A subgroup of the participants determined test–retest reliability (*n* = 46) and responsiveness (*n* = 27).

**Results:**

The ULFI-Ar construct validity obtained by the expletory factor analysis as one-factor structure, demonstrated an excellent test–retest reliability [intraclass correlation coefficient (ICC_2:1_) = 0.95], measurement error [standard error of measurement (SEM) = 4.43%; minimal detectable change at 90% confidence interval (MDC_90_) = 10.34%], medium internal responsiveness [Cohen’s *d* = 0.62 and standard response of mean (SRM) = 0.67], strong external responsiveness DASH-Arabic (r =—0.90; *p* < 0.001), and negative strong correlation with NPRS-Arabic (r =—0.75, *p* < 0.001).

**Conclusions:**

The ULFI-Ar is a valid, reliable, and responsive self-report questionnaire to assess UL-MSDs in Arabic speaking patients.

## Background

Musculoskeletal disorders (MSDs) are common complaints encountered by clinicians including physical therapists [1]. The upper limb MSDs (UL-MSDs) impact both health care resources and quality of life [1–3]. In Saudi Arabia, the prevalence of UL-MSDs in general population reaches up to 45.6% [2, 3].

One of the evaluation tools is self-reported outcome measures, which are designed to detect a patients’ health status, function level, and health-related quality of life [4, 5]. Furthermore, they measure people’s emotions, thoughts, behaviors, and circumstances associated with disability or impairment [6]. Several self-reported outcome measures have been developed for UL-MSDs including the Neck and Upper Limb Index (NULI) [7], Upper Extremity Functional Scale (UEFS) [8], Upper Extremity Functional Index (UEFI) [9], Disabilities of the Arm, Shoulder, and Hand (DASH) [10], QuickDASH [11], and QuickDASH-9 [12]. Most of these tools have limitations such as comprehensiveness, adequacy of the items towards the instrument domains, and generalization from a specific to general population [13, 14]. Other limitations are related to practical characteristics or interpretability [15, 16] and psychometric properties [9, 15].

The upper limb functional index (ULFI), on the other hand, has overcome the aforementioned limitations successfully. The ULFI has been used in several countries and translated and validated in many languages including Spanish [17], French-Canadian [18, 19], Turkish [20], Italian [21], Korean [22], Persian [23], Brazilian [24], Greek [25] and Urdu [26]. Since cultural background that may affect the original questionnaire, we recently translated and cross-culturally adapted the ULFI to Arabic language (ULFI-Ar). The ULFI-Ar demonstrated an excellent content validity (0.96) and high internal consistency (Cronbach’s α = 0.88) [27]. However, other psychometric measurements of the ULFI-Ar have not been studied. Thus, the current study aimed to test the longitudinal psychometric properties of the ULFI-Ar by investigating other measurements of validity and reliability, namely factorial validity, test–retest reliability, measurement error, minimal detectable change, and responsiveness. We hypothesized that the ULFI-Ar would have adequate construct validity, test–retest reliability, and responsiveness.

## Methods

This is an observational cross-sectional study that was conducted between of March and September 28, 2021 in (King Fahad Hospital for University in Al Khobar, Saudi Arabia). The Institutional Review Board of the (Imam Abdulrahman bin Faisal University) approved the study (IRB-PGS-2021–03-063; date: 22/02/2021). The study followed the guidelines of the Strengthening the Reporting of OBservational studies in Epidemiology (STROBE) [28].

### Participants

All participants were referred to the physical therapy department and recruited consecutively. The eligible criteria for recruitment were adult participants (18 to 60 years old), diagnosed with UL-MSDs including shoulder, elbow, wrist, or hand joints, and able to read and understand Arabic. Participants with any recent upper limb surgery, cognitive impairment, infectious disease, neurological disease, tumor, or other systematic diseases that could affect function of the upper limb were excluded. Further studies are needed to correlate between these factors (such as recent surgeries) and specific question item(s) are those are improper to ask. A written consent form was completed by each participant.

The recommended sample size is at least 5 times the number of the questionnaire items provided that the sample size is ≥ 100 participants [29]. Thus, 125 participants were required to achieve a statistical power of 80% for validation. To consider a dropout rate of 10%, 139 participants were consecutively recruited to complete the following questionnaires: ULFI-Ar, DASH-Arabic, and numeric pain rating scale (NPRS). The minimum number of participants recruited in previous research was 30 participants for test–retest reliability [19, 23] and 20 participants for responsiveness [19, 30].

### Measurement instruments

The ULFI is a single-page instrument with 25 items. It is a valid, reliable and responsive measure to assess people with UL-MSDs [22]. It has three-point response options of ‘Yes = 1’, ‘Partly = 0.5’, and ‘No = 0’ [31]. The total score ranges from 0 (maximum limitation) to 100 (full function), which can be calculating by the following equation: $[{\mathrm{ULFI}}_{\mathrm{Score}}=\{(\text{sum of the }25\text{ items points})\mathrm{\times}4\}-100]$. The ULFI permits up to two missing responses to validate scoring. The ULFI-Ar was equivalent to the English ULFI. In the ULFI-Ar, only a few items were adapted to fit the Arabic context. A more detailed description of the ULFI-Ar was previously reported [27]. The authors of the current study obtained permission from the authors of the original English ULFI to translate and validate the ULFI to Arabic.

The DASH-Arabic is divided into four sections: introduction, main 30 items, and two optional sections. The main 30 items target any functional level to the upper limb, the severity of symptoms, and psychosocial difficulties, whereas the optional sections address the work and sport impairments. Each statement has a five Likert scale response that ranges from 1 “without any difficulty or no symptoms exist” to 5 “unable to engage in activity or very severe symptoms”. A minimum of 27 items out of the main 30 must be answered to get the correct scoring. For scoring, the following formula is used: ${\mathrm{DASH}-\mathrm{Arabic}}_{100\mathrm{ score}}=\left\{\left(\frac{\mathrm{sum of completed responses}}{\mathrm{count of completed responses}}\right)-1\right\}\text{ x }25$. The higher the score, the higher the disability. The optional sections follow the same procedure but they require answering all five items. The DASH-Arabic is reliable, valid, and responsive [32, 33].

The NPRS-Arabic consists of a horizontal line of numerical point scale from 0 ‘no pain’ to 10 ‘extreme pain’. The participant was asked to rate the current pain intensity. The NPRS-Arabic is a valid, reliable, and responsive tool for pain intensity in UL-MSDs [34].

### Data and statistical analysis

Data was analyzed using IBM SPSS Statistics for Macintosh, Version 26.0. (IBM Corp. Armonk, NY, USA). The level of significance was set at *p* < 0.05. The mean and standard deviation (SD) were conducted as descriptive analysis for the demographic variables. The Shapiro–Wilk test was used to test data normality of the ULFI-Ar, DASH-Arabic, and NPRS-Arabic [35]. The data were normally distributed (*p* > *0.05)* for the ULFI-Ar. The DASH-Arabic and NPRS-Arabic demonstrated a relatively normal distribution for participants with elbow and wrist/hand disorders. However, the data distribution was inconsistent for pooled data and participants with shoulders disorders in both the DASH-Arabic and NPRS-Arabic. Paired t-test was performed to compare the scores of the ULFI-Ar, DASH-Arabic, and NPRS-Arabic for the test–retest and responsiveness in comparison with baseline. A ceiling or floor effect was determined if more than 15% of respondents revealed the highest or the lowest possible score, respectively [19].

Factor analysis was performed to evaluate construct validity of ULFI-Ar. Two classes of factor analysis were applied: Exploratory Factor Analysis (EFA) and Confirmatory Factor Analysis (CFA) [36]. Prior to the extraction of the factors, suitability of the respondent data was assessed by Kaiser–Meyer–Olkin (KMO) test with a value between 0.60 and 0.90 and a significant Bartlett’s Sphericity test [27]. The KMO result was 0.812 and the Bartlett’s sphericity test was significant (*p* < 0.001). Thus, these results confirmed factor analysis by using the EFA and CFA. The EFA was used with maximum likelihood extraction (MLE) and varimax rotation [37]. The factor extraction had three a-priori requirements: Eigenvalue > 1, accounting for > 10% of variance [38] and the ‘point of inflection’ on the scree plot [39]. The CFA was analyzed by using the IBM SPSS Amos 26.0.0 for Windows (Amos Development Corporation, Wexford, USA) to clarify the dimensions loading and the model fit. The fit indices were chi-square (χ^2^)/ degrees of freedom (DF), Root Means Square Error of Approximation (RMSEA), Comparative Fit Index (CFI), and Tucker-Lewis Index (TLI). These were considered adequate when χ^2^ / DF < 3, RMSEA < 0.10, CFI and TLI > 0.90, and a factor loading > 0.40 [40].

The test–retest reliability was assessed by interclass correlation coefficients [ICC_2,1_] [41] in a subgroup of the participants who completed the ULFI-Ar, DASH-Arabic, and NPRS-Arabic at two time intervals (baseline and 2–4 days) during non-treatment period. All participants were asked about their symptoms in the second interval to make sure that their symptoms were stable. The minimum accepted level of ICC for test–retest reliability was 0.70 [42]. The measurement error was expressed as the standard error of measurement (SEM) and calculated by using the following formula: $\mathrm{SEM}={\mathrm{SD}}_{(\mathrm{Baseline})} \sqrt{(1-\mathrm{ICC})}$, where SD_(Baseline)_ was standard deviation at baseline [35]. The minimal detectable change at 90% confidence interval (MDC_90_) was converted from SEM using the equation: ${\mathrm{MDC}}_{90}=\mathrm{SEM x }\sqrt{2}\mathrm{ x }1.65$ [35].

For responsiveness, another subgroup of the participants completed the three questionnaires twice: before treatment and after discharge, with a period of six weeks between these two tests. The responsiveness was determined by two methods. The internal responsiveness was assessed by the effect size (Cohen’s *d*) and the standard response mean (SRM) [35]. Cohen’s *d* can be obtained either by dividing the mean of pretest and posttest over standard deviation of both the baseline and post-treatment measurement ($d=\frac{\mathrm{mean}}{\mathrm{SD}}$) or by obtaining the paired-sample t-test on the square root of the sample size ($d=\frac{\mathrm{t}}{\sqrt{\mathrm{N}}}$). Both formulas reveal the same result. Cohen’s *d* is expressed as small (0.2), medium (0.5), and large (0.8) effect size [35]. The SRM was calculated by dividing the average difference between the baseline and responsiveness measurement over its standard deviation ($\mathrm{SRM }=\frac{{\mathrm{\rm X}}_{\mathrm{change}}}{{\mathrm{SD}}_{\mathrm{Xchange}}})$. The external responsiveness was computed by calculating the correlation between ULFI-Ar, DASH-Arabic, and NPRS-Arabic using Pearson’s correlation coefficients (*r*). A moderate external responsiveness (*r*) value is approximately 0.5 [29].

## Results

A total of 146 participants with UL-MSDs were screened. Three participants were excluded because they did not fulfill the inclusion criteria and four participants were excluded because of incomplete information. A total of 139 participants completed the ULFI-Ar, DASH-Arabic, and the NPRS-Arabic. Of these, 46 participants completed the same questionnaires for test–retest study and 27 patients for the responsiveness testing. Table 1 shows the demographic and clinical characteristics of the participants. Age of the participants was in the mid-thirties, and male participates were more than women. Average pain duration was 10 months and 57.6% of the participants had pain for more than 14 days. The most common affected joint was the shoulder with referred diagnosis of impingement and rotator cuff syndrome.

**Table 1:** Participants’ characteristics

	**Baseline (*n* = 139)**	**Test–retest (*n* = 46)**	**Responsiveness (*n* = 27)**
**Age (years)**^a^	38.67 ± 13.04	34.35 ± 10.96	33.56 ± 11.73
**Gender**			
*Male*	87 (62.6%)	28 (60.9%)	16 (59.3%)
*Female*	52 (37.4%)	18 (39.1%)	11 (40.7%)
**Occupational Status**			
*Student*	15 (10.8%)	8 (17.4%)	5 (18.5%)
*Employed*	85 (61.2%)	28 (60.9%)	19 (70.4%)
*Non-employed*	27 (19.4%)	8 (17.4%)	2 (7.4%)
*Retired*	12 (8.6%)	2 (4.3%)	1 (3.7%)
**Dominant hand**			
*Right*	125 (89.9%)	42 (91.3%)	23 (85.2%)
*Left*	14 (10.1%)	4 (8.7%)	4 (14.8%)
**Pain duration (weeks)**	47.81 ± 43.50	42.43 ± 58.43	41.43 ± 52.28
**Diagnosis**			
**Shoulder**	76 (54.6%)	20 (43.5%)	16 (59.3%)
*Adhesive capsulitis*	15 (10.8)	6 (13.0)	1 (3.7)
*Impingement*	21 (15.1)	7 (15.2)	7 (25.9)
*Rotator Cuff Syndrome*	26 (18.7)	7 (15.2)	7 (25.9)
*Instability*	14 (10.1)	0	1 (3.7)
**Elbow**	16 (11.5%)	8 (17.4%)	3 (11.1%)
*Tendinitis*	8 (5.8)	4 (8.7)	2 (7.4)
*Fractures*	8 (5.8)	4 (8.7)	1 (3.7)
**Wrist & Hand**	25 (18.0%)	12 (26.1%)	5 (18.5%)
*Fractures*	19 (13.7)	11 (23.9)	5 (18.5)
*Trigger finger*	6 (4.3)	1 (2.2)	0
**Multiple joints**	15 (10.8%)	2 (4.3%)	1 (3.7%)
**Other**^b^	7 (5.0)	4 (8.7)	2 (7.4)
**Chronicity**			
*Acute (0–4 days)*	32 (23.0%)	6 (13.0%)	10 (37.0%)
*Sub-acute (5–14 days)*	27 (19.4%)	13 (28.3%)	7 (25.9%)
*Chronic (*> *14 days)*	80 (57.6%)	27 (58.7%)	10 (37.0%)
**Work-related injury**			
*Yes*	9 (6.5%)	2 (4.3%)	1 (3.7%)
*No*	121 (87.1%)	41 (89.1%)	25 (92.6%)
*Don't Know*	9 (6.5%)	3 (6.5%)	1 (3.7%)
**Post-surgery**			
*Yes*	35 (25.2%)	n/a	8 (29.6%)
*No*	104 (74.8%)	46 (100%)	19 (70.4%)

^a^ All data are expressed in frequency (percentage), except age and pain duration are expressed in mean ± standard deviation

^b^ Carpal tunnel syndrome, bone inflammation, biceps tendinitis, hand extensors reconstruction

Table 2 presents the mean and standard deviation obtained from the three questionnaires, which showed no floor or ceiling scores. There were no missing responses for the ULFI-Ar. The ‘Half’ response option was used by 95% of the participants in a total of 22% of their responses. The DASH-Arabic had missing responses from 26 different items from 84 (60.4%) participants. Six participants had ≥ 3 missing responses in completing the DASH-Arabic; therefore, they were excluded from the data analysis.

**Table 2:** The scores of the questionnaires for the baseline, test–retest, and responsiveness

	Baseline		Test–retest		Responsiveness	
Questionnaires	n (RR)	Mean ± SD (range)	n (RR)	Mean ± SD (range)	n (RR)	Mean ± SD (range)
ULFI-Ar	139 (100%)	59.91 ± 18.9 (2–98)	46 (100%)	64.30 ± 21.41 (22–100) *(p* = *0.49)*^a^	27 (100%)	76.00 ± 19.49 (24–100) *(p* = *0.002)*^b^
DASH-Arabic	133 (95.7%)	27.33 ± 17.19 (0–74)	41 (95.4%)	25.75 ± 17.75 (0–73.33) (*p* = *0.92)*^a^	26 (96.3%)	18.12 ± 19.58 (0–91.38) *(p* = *0.02)*^b^
NPRS-Arabic	139 (100%)	4.6 ± 2.75 (0–10)	46 (100%)	3.3 ± 3.28 (0–10) *(p* = *0.67)*^a^	27 (100%)	2.9 ± 2.79 (0–8) *(p* = *0.02)*^b^

*DASH* Disabilities of Arm, Shoulder, and Hand, *NPRS* Numeric Pain Rating Scale, *RR* Response Rate, *SD* Standard Deviation, *ULFI-Ar* Upper Limb Functional Index-Arabic

^a^ Difference between baseline and test–retest

^b^ Difference between baseline and responsiveness

**Fig. 1:**
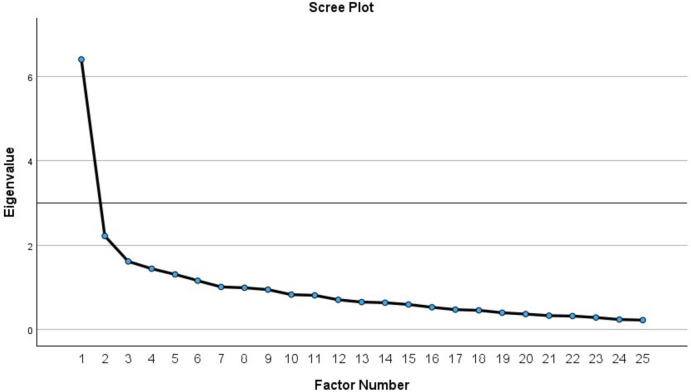
Scree plot of the one factor of the upper limb functional index—Arabic

**Fig. 2:**
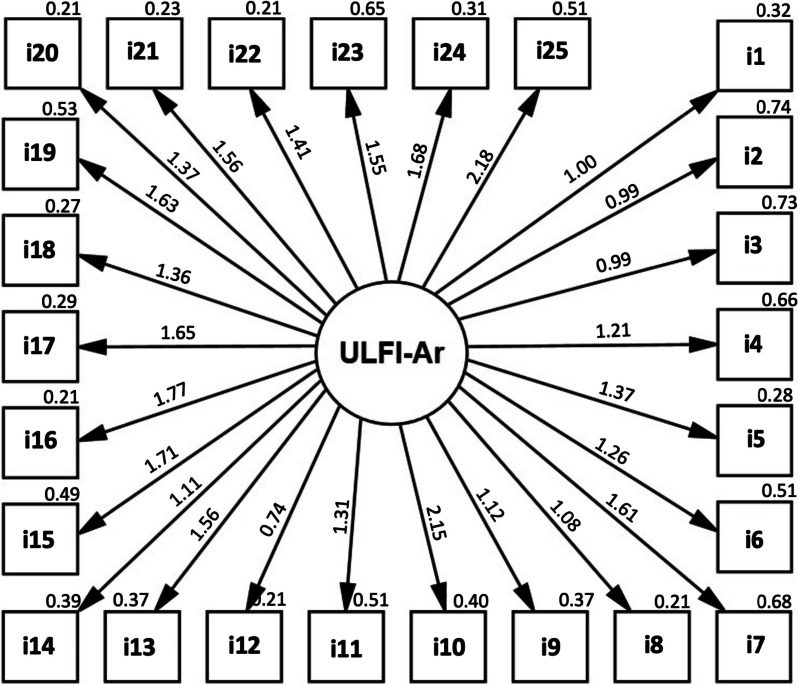
Confirmatory factor analysis and standardized factor loading values of the upper limb functional index—Arabic

For construct validity, the EFA revealed six factors with Eigenvalues > 1; where only one factor exceeded 10% variance (25.62%) and was presented before the inflection point (Fig. 1). As the three priori criteria were met, this result indicated a unidimensional structure of the tool. Table 3 shows the items factor loading for the one-factor solution and its average scores for each item. For factor loading, eight items scored below 0.50 (lowest = 0.34), while no items scored > 0.80 (highest = 0.72), which indicated no item redundancy. The extraction component under the item average score showed only three items had scores below 0.50 (lowest = 0.33), expressing a strong distinct component. The unidimensional factor was analyzed with CFA and showed that all 25 items factor loading was more than 0.40 (Fig. 2). Fit model of the CFA was acceptable [df = 275, χ^2^ = 588.98 (*p* < 0.001), χ^2^ / df = 2.14, CFI = 0.652, RMSEA = 0.091, and TLI = 0.620], which supported that the 25 items structure should be reserved.

**Table 3:** Factor analysis loading for the upper limb functional index – Arabic

Item		Factor loading	Item average score ^a^
1	I stay at home most of the time	0.340	0.344
2	I change position frequently for comfort	0.529	0.598
3	I avoid heavy jobs e.g. cleaning, lifting more than 5 kg or 10lbs, gardening, etc	0.431	0.655
4	I rest more often	0.514	0.538
5	I get others to do things for me	0.567	0.585
6	I have the pain / problem almost all the time	0.537	0.605
7	I have difficulty lifting and carrying (e.g. bags, shopping up to 5 kg or 10lbs)	0.580	0.618
8	My appetite is now different	0.429	0.542
9	My walking or normal recreation or sporting activity is affected	0.445	0.392
10	I have difficulty with normal home or family duties and chores	0.722	0.582
11	I sleep less well	0.472	0.493
12	I need assistance with personal care e.g. washing and hygiene	0.372	0.653
13	My regular daily activities (work, social contact) are affected	0.579	0.548
14	I am more irritable and / or bad tempered	0.462	0.587
15	I feel weaker and / or stiffer	0.630	0.598
16	My transport independence is affected (driving, public transport)	0.604	0.627
17	I have difficulty putting my arm into a shirt sleeves or need assistance dressing	0.566	0.682
18	I have difficulty writing or using a key board and / or ‘mouse’	0.488	0.527
19	I am unable to do things at or above shoulder height	0.620	0.643
20	I have difficulty eating and / or using utensils (e.g. knife, fork, spoon, chop sticks)	0.503	0.652
21	I have difficulty holding and moving dense objects (e.g. mugs, jars, cans)	0.614	0.656
22	I tend to drop things and / or have minor accidents more frequently	0.520	0.568
23	I use the other arm more often	0.544	0.521
24	I have difficulty with buttons, keys, coins, taps / faucets, containers or screw-top lids	0.590	0.645
25	I’ve difficulty opening, holding, pushing or pressing (eg. triggers, levers, heavy doors …)	0.700	0.674

^a^ Extraction methods by maximum likelihood extraction (MLE)

Paired t-tests showed no significant difference between the ULFI-Ar testing and retesting scores (t = 0.695; *p* = 0.49). The test–retest reliability of the ULFI-Ar was excellent (ICC_2,1_ = 0.95) with an individual range of 95% CI = 0.90 – 0.97. The measurement error from the SEM and MDC_90_ were 4.43% and 10.34%, respectively.

The internal responsiveness of the ULFI-Ar as represented by the paired t-test resulted in significant difference between the baseline and responsiveness scores (t = 3.47; *p* = 0.002). The effect size was medium (Cohen’s *d* = 0.67; 95% CI = 1.08 – 1.06) and SRM was also medium (0.667; 95% CI = 0.24 – 0.98). The percentage difference between SRM and effect size for the same change measurement on the same participant was 1%. The external responsiveness was strongly correlated with the DASH-Arabic (r =—0.90). A negative strong correlation was found between the ULFI-Ar and NPRS-Arabic (r =—0.75, *p* < 0.001).

Table 4 summarizes the psychometric characteristics of the three questionnaires including reliability, validity, and responsiveness.

**Table 4:** Methodological characteristics of, upper limb functional index-Arabic, disabilities of arm, shoulder, and hand, and numeric pain rating scale

Outcome measure	Reliability			Responsiveness			
	Test–retest	Error score		Internal			External
	ICC_2,1_	SEM	MDC_90_	SD	ES	SRM	r
ULFI-Ar	0.95	4.43	10.34	18.90	0.67	0.67	0.90
DASH-Arabic	0.89	5.70	13.30	17.19	0.64	n/a	0.90
NPRS-Arabic	0.82	1.26	2.93	2.96	0.37	n/a	0.75

*DASH* Disabilities of Arm, Shoulder, and Hand, *ES* Effect Size, *ICC* Intraclass Correlation Coefficient, *MDC* Minimal Detectable Change, *n/a* not applicable, *NPRS* Numeric Pain Rating Scale, *r* Pearson’s (r) correlation coefficient, *SD* Standard Deviation, *SRM* Standard Response Mean, *SEM* Standard Error of Measurement, *ULFI-Ar* Upper Limb Functional Index-Arabic

## Discussion

The psychometric properties testing demonstrated adequate results that support the validity, reliability, and responsiveness of the ULFI-Ar. The construct validity of the ULFI-Ar in the current study was supported by the single-factor solution that emerged from the factor analysis. Although six factors had Eigenvalue > 1.0, only one factor accounted for > 10% of variance (29.4%). This result is in agreement with the studies of the English [31], Spanish [17], and Persian [23] versions. Conversely, the Turkish, Greek, and Urdu studies found that two factors showed variance > 10% from six to seven factors with Eigenvalues > 1.0 [20, 25, 26]. The Brazilian version used a parallel analysis as an alternative method and confirmatory factor analysis (CFA), which both extracted only one factor [24]. The other studies did not report factor analysis results [18, 22]. The Italian version used a sample size lower than the required participants [17]. In the current study, there were 8 items that were scored below 0.50 in the factor loading compared with the Spanish [5 items] [17], Greek [5 items] [25] Urdu [7 items] [26], Turkish [9 items] [20], Persian [10 items] [23], and English version [14 items] [31]. This finding suggests that reduction of the total number of items may reduce the respondent burden and improve the tool practicality [16]. In our study, no items scored > 0.80 (highest = 0.72) which confirms no item redundancy. In the extraction component, only two items were below 0.50 (lowest = 0.34), suggesting a strong distinct component for upper limb outcome measure. In our study, the CFA testing of the unidimensional model of the ULFI-Ar showed a factor loading more than 0.40 for all the 25 items. This is in agreement with the Brazilian study [24]. However, in the current study, both the CFI (0.652) and TLI (0.620) were less than the recommended levels (> 0.090). These low values may be resolved by increasing the sample size to at least 200 participants although a minimum of 100 participants was accepted for factor analysis [36]. The ULFI-Ar has a greater value of χ^2^ / df [2.14] and RMSEA [0.091] than the Brazilian version [1.75 and 0.063, respectively] [24]. However, ULFI-Ar demonstrated lower values of the CFI [0.652] and TLI [0.620] compared with the Brazilian version [0.918 and 0.910, respectively] [24].

The high test–retest reliability of the ULFI-Ar (ICC_2:1_ = 0.95) supports the instrument’s stability. This is comparable with the English [ICC_2:1_ = 0.98] [31], Greek [ICC_2:1_ = 0.97] [25], Italian [ICC_2:1_ = 0.94] [21], Spanish [ICC_2:1_ = 0.93] [17], Persian [ICC_2:1_ = 0.93] [23], French-Canadian [ICC_2:1_ = 0.92] [19], Urdu [ICC_2:1_ = 0.91] [26], Korean [ICC_2:1_ = 0.90] [22], and Brazilian versions [ICC_2:1_ = 0.90] [24]; but higher than the Turkish version [ICC_2:1_ = 0.72] [20]. The authors of the Turkish version contributed the lower value of test–retest reliability in their study to that all participants reported the ‘same’ on ‘global rating of change’ [20]. We do not agree with the authors, as reporting “the same” by the participants indicates that their status was stable, and consequently, the ICC value should be higher.

Measurement error and sensitivity determined from SEM and MDC_90_ were 4.43% and 10.34%, respectively. The small value of the SEM in this study suggests a good measure of precision [35]. This SEM is comparable to the Greek [3.34%] [25], English [3.41%] [31], Urdu [3.89%] [26], French-Canadian [4%] [18], Turkish [2.95%] [20], Persian [3.11%] [23], and Spanish [3.52%] [17]; but lower than the Brazilian version [6.11] [24]. The MDC_90_ in other versions were: 5.53% (Turkish) [20], 7.25% (Persian) [23], 7.79% (Greek [25], 7.93 (English) [31], 8.03% (Spanish) [17], 9.3% (French-Canadian) [19], 10.6% (Urdu) [26], 12% (Italian) [21], and 14.26% (Brazilian) [24].

Internal responsiveness measured by Cohen’s *d* effect size (0.67) and SRM (0.67) was moderate. Our finding is similar to the French-Canadian version [*d* = 0.62, SRM = 0.88] [19] but lower than the Greek and English versions [*d* = 1.19 and 0.93, SRM = 1.31 and 1.33, respectively] [25, 31]. External responsiveness of the ULFI-Ar was strong as estimated by Pearson’s correlation coefficients with the DASH-Arabic (r = 0.90) and the NPRS-Arabic (r = 0.75). In comparison, only the French-Canadian study (r =—0.64) investigated this type of responsiveness in relation to the DASH-FC [19]. In both studies, the Arabic and French-Canadian, the time interval between the two measurements ranged from 2 to 6 weeks and showed a significant difference between the baseline and responsiveness readings as detected by paired *t*-test. It is an optimal period for the clinician to detect the patients’ functional status in a short time and to evaluate the intervention outcome [19].

The main strength of this study is that we attempted to investigate all psychometric properties of the ULFI-Ar.. Another strength is that our study recruited participants with acute, subacute, and chronic conditions [17]. A limitation may be that sample recruitment was from one clinical setting. Although the standard Arabic language was used in the translation of the ULFI, inclusion of Arabic participants other than Saudi could confirm conflicting findings. Moreover, sample size was not calculated for reliability and responsiveness although we tried to recruit more than the number of participants used in previous similar research. In addition, the current study did not include an assessment of the psychometric properties of the ULFI-Ar for patients undergoing other treatments than physical therapy, which in turn may limit the breadth of the study.

## Conclusion

The study showed that the ULFI-Ar is a unidimensional factor and has excellent test–retest reliability, and medium to strong responsiveness. The ULFI-Ar can be used as an appropriate outcome measure in clinical and research setting for Arabic speaking patients with UL-MSDs. Future research is recommended to assess the psychometric properties of the ULFI-Ar in patients undergoing treatments than physical therapy.

## Data Availability

The datasets used and/or analysed during the current study are available from the corresponding author on reasonable request.

## Abbreviations

CFA: Confirmatory factor analysis
CFI: Comparative Fit Index
DAHS: Disability of arm, hand and shoulder
df: Degrees of freedom
EFA: Exploratory factor analysis
ICC: Interclass correlation coefficient.
KMO: Kaiser-Meyer-Olkin
MDC: Minimal detectable change
MSD: Musculoskeletal disorder
NPRS: Numeric pain rating scale
NULI: Neck and upper limb index
RMSEA: Root Mean Square Error of Approximation
SD: Standard deviation
SEM: Standard error of measurement
SRM: Standard response mean
STROBE: Strengthening the reporting of observational studies in epidemiology
TLI: Tucker Lewis Index
UEFI: Upper extremity functional index
UEFS: Upper extremity functional scale
UL-MSD: Upper limb musculoskeletal disorder
ULFI: Upper limb functional index
ULFI-Ar: Upper limb functional index—Arabic

## References

[1] Govaerts R, Tassignon B, Ghillebert J, Serrien B, De Bock S, Ampe T (2021). Prevalence and incidence of work-related musculoskeletal disorders in secondary industries of 21st century Europe: a systematic review and meta-analysis. BMC Musculoskelet Disord.

[2] Algarni AD, Al-Saran Y, Al-Moawi A, Bin Dous A, Al-Ahaideb A, Kachanathu SJ. The Prevalence of and Factors Associated with Neck, Shoulder, and Low-Back Pains among Medical Students at University Hospitals in Central Saudi Arabia. Pain Res Treat. 2017;2017:7. 10.1155/2017/1235706 PMC5697379 29238618

[3] Almomani F, Alghwiri AA, Alghadir AH, Al-momani A, Iqbal A (2019). Prevalence of upper limb pain and disability and its correlates with demographic and personal factors. J Pain Res.

[4] Morris LA, Miller DW (2002). The Regulation of Patient-Reported Outcome Claims: Need for a Flexible Standard. Value Health.

[5] Sloan JA, Dueck A, Qin R, Wu W, Atherton PJ, Novotny P (2008). Quality of life: The assessment, analysis, and interpretation of patient-reported outcomes. Biometrics.

[6] Jayakumar P, Williams M, Ring D, Lamb S, Gwilym S. A systematic review of outcome measures assessing disability following upper extremity trauma. J Am Acad Orthop Surg Glob Res Rev. 2017;1(4):e021. 10.5435/JAAOSGlobal-D-17-00021 PMC6132302 30211355

[7] Stock SR, Streiner D, Reardon R, Darzins S, Dilworth P, Tugwell P (1995). The impact of neck and upper limb musculoskeletal disorders on the lives of affected workers: Development of a new functional status index. Qual Life Res.

[8] Pransky G, Feuerstein M, Himmelstein J, Katz JN, Vickers-Lahti M (1997). Measuring functional outcomes in work-related upper extremity disorders: development and validation of the upper extremity function scale. J Occup Environ Med.

[9] Stratford PW, Binkley JM, Stratford DM (2001). Development and initial validation of the upper extremity functional index. Physiother Can.

[10] Beaton DE, Katz JN, Fossel AH, Wright JG, al et. Measuring the whole or the parts? Validity, reliability, and responsiveness of the disabilities of the arm, shoulder and hand outcome measure in different regions of the upper extremity. J Hand Ther. 2001;14(2):128–46. 11382253

[11] Beaton D, Wright J, Katz J (2005). Development of the QuickDASH: Comparison of three Item-reduction approaches. J Bone Joint Surg.

[12] Gabel CP, Yelland M, Melloh M, Burkett B (2009). A modified QuickDASH-9 provides a valid outcome instrument for upper limb function. BMC Musculoskelet Disord.

[13] Gabel P. Development and initial validation of a new regional outcome measure: the Upper Limb Disability Questionnaire (ULDQ) [Thesis]. Australia: Northern Territory University; 2003.

[14] Devereux JJ, Vlachonikolis IG, Buckle PW (2002). Epidemiological study to investigate potential interaction between physical and psychosocial factors at work that may increase the risk of symptoms of musculoskeletal disorder of the neck and upper limb. Occup Environ Med.

[15] Michener LA, Leggin BG (2001). A review of self-report scales for the assessment of functional limitation and disability of the shoulder. J Hand Ther.

[16] Amadio PC (2001). Outcome assessment in hand surgery and hand therapy: An update. J Hand Ther.

[17] Cuesta-Vargas AI, Gabel PC (2013). Cross-cultural adaptation, reliability and validity of the Spanish version of the upper limb functional index. Health Qual Life Outcomes.

[18] Hamasaki T, Demers L, Filiatrault J, Aubin G (2014). A cross-cultural adaptation of the Upper Limb Functional Index in French Canadian. J Hand Ther.

[19] Hamasaki T, Demers L, Filiatrault J (2015). Test–retest reliability and responsiveness of a French Canadian Upper Limb Functional Index (ULFI-FC). Disabil Rehabil.

[20] Tonga E, Durutürk N, Gabel PC, Tekindal A (2015). Cross-cultural adaptation, reliability and validity of the Turkish version of the Upper Limb Functional Index (ULFI). J Hand Ther.

[21] Sartorio F, Moroso M, Vercelli S, Bravini E, Medina ME, Spalek R (2015). Cross-cultural adaptation, and validity of the italian version of the upper limb functional index (ULFI-I). G Ital Med Lav Ergon.

[22] In TS, Jung JH, Kim KJ, Lee CR, Jung KS, Cho HY (2017). The reliability and validity of the Korean version of the Upper Limb Functional Index. J Phys Ther Sci.

[23] Mokhtarinia HR, Zareiyan A, Gabel CP (2021). Cross-cultural adaptation, validity, and reliability of the Persian version of the Upper Limb Functional Index. Hand Ther.

[24] Takahasi HY, Fidelis-de-Paula-Gomes CA, Gabel CP, Dibai-Filho AV (2021). Translation, cross-cultural adaptation and validation of the Upper Limb Functional Index (ULFI) into Brazilian Portuguese in patients with chronic upper limb musculoskeletal disorders. Musculoskelet Sci Pract.

[25] Chamogeorgakis G, Karanasios S, Theotokatos G, Vasilogeorgis I, Korakakis V. Cross-Cultural Adaptation and Measurement Properties of the Upper Limb Functional Index (ULFI) for Greek-Speaking Patients. Cureus. 2023; Available from: https://www.cureus.com/articles/158489-cross-cultural-adaptation-and-measurement-properties-of-the-upper-limb-functional-index-ulfi-for-greek-speaking-patients. [cited 2023 Sep 12]. 10.7759/cureus.40029 PMC10323981 37425611

[26] Arooj A, Amjad F, Tanveer F, Arslan AU, Ahmad A, Gilani SA (2022). Translation, cross-cultural adaptation and psychometric properties of Urdu version of upper limb functional index; a validity and reliability study. BMC Musculoskelet Disord.

[27] Albahrani YA, Alshami AM (2022). Cross-cultural adaptation of the upper limb functional index in Arabic. Acta Biomedica Atenei Parmensis.

[28] Cuschieri S (2019). The STROBE guidelines. Saudi J Anaesth.

[29] Mokkink LB, Terwee CB, Patrick DL, Alonso J, Stratford PW, Knol DL (2010). The COSMIN checklist for assessing the methodological quality of studies on measurement properties of health status measurement instruments: an international Delphi study. Qual Life Res.

[30] Gabel CP, Michener LA, Burkett B, Neller A (2006). The Upper Limb Functional Index: Development and determination of reliability, validity, and responsiveness. J Hand Ther.

[31] Gabel CP MSc, Michener LA PhD, PT, ATC, Melloh M MD, MPH, Burkett B PhD. Modification of the Upper Limb Functional Index to a three-point response improves clinimetric properties. J Hand Ther. 2010;23(1):41–52. 10.1016/j.jht.2009.09.007 19963344

[32] Alotaibi NM (2010). Cross-cultural adaptation process and pilot testing of the Arabic version of the Disability of the Arm, Shoulder and Hand (DASH-Arabic). Hand Therapy.

[33] Alotaibi NM, Aljadi SH, Alrowayeh HN (2016). Reliability, validity and responsiveness of the Arabic version of the Disability of Arm, Shoulder and Hand (DASH-Arabic). Disabil Rehabil.

[34] Alghadir AH, Anwer S, Iqbal ZA (2016). The psychometric properties of an Arabic numeric pain rating scale for measuring osteoarthritis knee pain. Disabil Rehabil.

[35] Streiner DL, Norman GR, Cairney J. Health measurement scales: a practical guide to their development and use. Fifth. Oxford: Oxford University Press; 2015.

[36] Williams B, Onsman A, Brown T (2010). Exploratory Factor Analysis: A Five-Step Guide for Novices. Australasian Journal of Paramedicine.

[37] Watkins MW (2018). Exploratory Factor Analysis: A Guide to Best Practice. J Black Psychol.

[38] de Vet HCW, Adèr HJ, Terwee CB, Pouwer F (2005). Are factor analytical techniques used appropriately in the validation of health status questionnaires? A systematic review on the quality of factor analysis of the SF-36. Qual Life Res.

[39] Sarstedt M, Mooi E. Factor Analysis. In: Sarstedt M, Mooi E, editors. A Concise guide to market research: The process, data, and methods using IBM SPSS Statistics. Berlin: Springer Berlin Heidelberg; 2014. p. 235–72.

[40] Shek DT, Yu L (2014). Confirmatory factor analysis using AMOS: a demonstration. Int J Disabil Hum Dev.

[41] Koo TK, Li MY (2016). A Guideline of Selecting and Reporting Intraclass Correlation Coefficients for Reliability Research. J Chiropr Med.

[42] van Kampen DA, Willems W, van Beers LWAH, Castelein RM, Scholtes VAB, Terwee CB (2013). Determination and comparison of the smallest detectable change (SDC) and the minimal important change (MIC) of four-shoulder patient-reported outcome measures (PROMs). J Orthop Surg Res.

